# Mining ship deficiency correlations from historical port state control (PSC) inspection data

**DOI:** 10.1371/journal.pone.0229211

**Published:** 2020-02-21

**Authors:** Junjie Fu, Xinqiang Chen, Shubo Wu, Chaojian Shi, Huafeng Wu, Jiansen Zhao, Pengwen Xiong

**Affiliations:** 1 Merchant marine college, Shanghai Maritime University, Shanghai, China; 2 Institute of Logistics Science and Engineering, Shanghai Maritime University, Shanghai, China; 3 School of Information Engineering, Nanchang University, Nanchang, China; Central South University, CHINA

## Abstract

Early warning on the ship deficiency is crucial for enhancing maritime safety, improving maritime traffic efficiency, reducing ship fuel consumption, etc. Previous studies focused on the ship deficiency exploration by mining the relationships between the ship physical deficiencies and the port state control (PSC) inspection results with statistical models. Less attention was paid to discovering the correlation rules among various parent ship deficiencies and subcategories. To address the issue, we proposed an improved Apriori model to explore the intrinsic mutual correlations among the ship deficiencies from the PSC inspection dataset. Four typical ship property indicators (i.e., ship type, age, deadweight and gross tonnage) were introduced to analyze the correlations for the ship parent deficiency categories and subcategories. The findings of our research can provide basic guidelines for PSC inspections to improve the ship inspection efficiency and maritime safety.

## 1. Introduction

Port state control (PSC) inspection is performed to ensure maritime traffic safety, improve the maritime efficiency, etc. The censored ship will be obligatorily detained and repaired when the PSC inspection results demonstrate that the ship is unqualified for sailing on waterway channels. The PSC inspection data provide us unique ship hull deficiency, which helps maritime traffic participants take early-warning measurements to avoid potential maritime accidents. More specifically, several studies have been conducted to explore the intrinsic relationship between the inspection PSC dataset and the ship detention cases (i.e., learning ship detention patterns from the PSC inspection data) [1–3]. Previous studies suggested that holistic PSC inspection (usually implemented by maritime safety administration) is crucial for finding the inconspicuous but fatal ship deficiencies, which significantly reduces maritime accidents [4].

Many studies attempted to analyze the intrinsic correlation between the ship detention incidents and large-scale PSC inspection data (by deeply mining ship detention information). More specifically, many scholars deeply explored PSC inspection code data (i.e., ship deficiency types) to extract the intrinsic ship deficiency identification rules by aggregating the ship detention codes. Wang et al. analyzed the oil tanker detention possibility by diving into the PSC inspection deficiency data [5]. Maria et al. established a Bayesian network model to explore the relationship between ship data (e.g., age, type, gross tonnage) and maritime accidents, and they quantitatively identified the effects of the ship deficiency on the maritime accidents [6]. Zhang et al. employed the principal component analysis method to analyze the PSC inspection data and obtained the main ship detention list, which deserves PSC officials special inspection attention [7,8].

Several studies focused on analyzing previous maritime accident datasets to collect the PSC priority list, which helps the maritime traffic participants take early-warning activities (e.g., rectifying deficiency at the next port or in two weeks) to avoid maritime traffic incidents. Yu et al. established a maritime accident database with snowflake schema, and an improved Apriori algorithm was developed to determine the intrinsic ship deficiency correlations [9]. By considering the human being interferences, Kokotos et al. attempted to obtain the main maritime accident factors following the international security management (ISM) rules [10]. Weng et al. used the association rules to determine crucial maritime accident contributory factors and mutual coupled effects on the ship detention incident [11]. Similar studies can be found in [12–19].

It is noted that the majority of previous studies focused on the correlation analysis between ship physical deficiencies and PSC detection results (e.g., fire-fighting equipment malfunction and incomplete ship-borne facility maintenance log). Little attention was paid to exploring the correlations between the collected ship inspection deficiency dataset and the PSC data using statistical analysis logics. The main difficulty is that there are many categories, which consist of different defective subcategories, and the ship samples are insufficient for further analysis. More specifically, the maritime regulation officials indicate that there are at least 568 types of defective subcategories for PSC inspection data, and several ship deficiency data have been masked due to data sensitivity. To address the issue, we proposed an improved Apriori algorithm by discarding trivial PSC inspection data to recognize implicit relationships between the ship deficiencies and the inspection data (DQCPEA).

Our contributions can be described as follows: (1) we performed the statistical analysis on the ship detention cases with the historical PSC data and thus identified the ship deficiency types that are closely related to the ship detention events; (2) we proposed an improved Apriori model to quantify the correlations among various ship deficiencies of triggering ship detention events; (3) we qualitatively explored the ship detention risk from the perspective of ship profiles (i.e., parent deficiency) and ship subcategory deficiencies (i.e., child deficiencies). The remainder of this paper is structured as follows. Section 2 explains the fundamental terms and framework for correlation identification from the ship inspection data. Section 3 discusses the ship deficiency recognition results with ship inspection data. Section 4 briefly concludes this study and provides potential future research directions.

## 2. Model development

The correlation analysis has shown its success in exploring intrinsic feature exploration applications (including the maritime knowledge discovery). More specifically, previous studies suggested that ship deficiencies were highly related in triggering ship detention events according to the PSC detection reports [20]. In addition, to find the rules conducted in the PSC inspection activities, we interviewed many maritime practitioners (i.e., maritime officials) in the form of a questionnaire, which is consistent with literature reviewing results. Stimulated by those results, we conducted a correlation analysis on mining the historical PSC inspection data. The Apriori relevant models have been employed to identify the crucial ship deficiencies from different ship detention cases, indicating favorable ship deficiency recognition results [20,21]. In that manner, the Apriori model is considered as an efficient model to tackle the challenge of exploiting intrinsic ship deficiency categories from the historical PSC dataset.

### 2.1 Basic concepts

The association rule is commonly used to mine the implicit correlation from large-scale samples. More specifically, the association rule aims to quantitatively analyze different mutual effects, which is applicable to explore the implicit PSC inspection data knowledge. The following terms are first illustrated to help the readers easily understand our proposed framework [22]. Several maritime terminologies used in our study were referred to [23].

**Item:** item is the basic and inseparable unit in the PSC inspection database. More specifically, the item is a ship deficiency category, which is denoted as *k* in our study.

**Itemset:** the term itemset is a collection of items, which is denoted as *i*_*k*_. A collection of itemsets is presented as *I* = {*i*_1_, *i*_2_, ⋯, *i*_*k*_}. Note that the itemset is the ship deficiency collection.

**Transaction:** a transaction is a collection of to-be processed items for a basic PSC database operation (i.e., add, delete, insert and query operations) and denoted as *T*. The transaction is considered a subset of *T* when itemset *I* contains all deficiencies in the PSC inspection database.

**Itemset frequency:** suppose that we have PSC inspection itemset *X* = {*x*_1_, *x*_2_, ⋯, *x*_*n*_} and transaction set *Y* = {*T*_1_, *T*_2_, ⋯, *T*_*m*_}, the PSC transaction itemset frequency *P*(*X*) is as follows:

$$P(X)=P(x_{1}\cap x_{2}\cap \cdots \cap x_{n})=\left|X\right|/\left|Y\right|$$ (1)

where |*X*| is the PSC inspection number for itemset *X*, and number *n* is the item sequence. Symbol |*Y*| is the transaction number.

**Support level:** the support level is the ratio between the ship deficiency transaction intersection of *T*(*A*) and *T*(*B*), which is denoted as Sup(*A* ⇒ *B*):

$$Sup(A\Rightarrow B)=P(AB)=\frac{a}{c}$$ (2)

where *P*(*AB*) is the frequency that both itemsets A and B simultaneously occur during the ship inspection procedure. The number of ships with deficiency types A and B is denoted as *a*, and *c* is the overall number of PSC inspected ships.

**Confidence level:** the confidence level reflects the probability of identifying ship deficiency B when a type-A ship deficiency occurs. Confidence level Conf(*A* ⇒ *B*) reveals the ratio between the number of ships involving deficiencies A and B in the PSC inspection database and the number of ships with deficiency category A. Conf(*A* ⇒ *B*) is denoted as follows:

Conf(A⇒B)=Sup(A⇒B)/Sup(A)=P(B|A) (3)

*P*(*B*|*A*) is a conditional probability formula, which is reformulated as *P*(*B*|*A*) = *P*(*AB*)/*P*(*A*).

### 2.2 DQCPEA algorithm for identifying ship deficiency

Currently, there are many algorithms to explore the association rules, but most of them are optimized based on the Apriori algorithm, such as the Partition algorithm, Sampling algorithm, and FP-growth algorithm [24–26]. More specifically, both Partition algorithm and Sampling algorithm are used to improve the Apriori algorithm efficiency by optimizing the data scan frequency. For example, the Partition algorithm is used to mine the frequent itemset by evenly dividing the initial database, but the results are not perfectly accurate [27–31]. The sampling algorithm obtains the frequent itemset identification with a similar logic to that of the Partition algorithm. The FP-growth algorithm generates a candidate frequent itemset by applying the FP-tree data structure. The main weakness of the FP-growth algorithm is the large computation cost, and some frequent items may be missed due to sampling error [32–35].

Compared to typical association rule exploration models, the Apriori relevant algorithms, which serve as typical association rule exploration techniques, show high performance in exploring Boolean association rules by mining from the frequent itemset data. The Apriori model employs the iterative search method to fulfill the frequent itemset identification task, which improves the search efficiency of frequent itemsets with the a priori property criterion. Indeed, the main idea of the Apriori model is to generate a bank of itemset candidates, which are further processed by the frequent itemset evaluation procedure. More specifically, the Apriori algorithm employs the layer-nesting iterative search logic to implement the task of frequent itemset search. The core idea of the Apriori algorithm is to generate a set of candidate itemsets, calculate the evaluation metric parameters (i.e., support and confidence) by exploring the dataset, and further determine whether the candidate itemset belongs to a frequent itemset. More details can be found in [36]. The Apriori algorithm assumes that the nonempty subsets of a frequent itemset are frequent itemsets, which greatly reduces the model computational consumption, [29,37–39]. Several itemset types of PSC inspection data are null (i.e., the ship deficiency information is deliberately discarded due to data sensitivity). Thus, the traditional Apriori algorithm cannot be easily used to explore the association rules from the PSC inspection data. To address this issue, we propose a data quality control procedure enhanced Apriori algorithm (DQCPEA) to suppress the null ship deficiency from the PSC inspection data sources. More specifically, the ship deficiency categories with a few inspection samples are iteratively discarded to avoid being further analyzed. The pseudo code for the proposed Apriori algorithm is as follows:

**Algorithm 1** data quality control procedure enhanced Apriori algorithm (DQCPEA)

**Input:** PSC inspection data set, minimum support threshold *α*, and minimum support threshold *β*.

**Output:** frequent ship deficiency itemsets and the corresponding support and confidence.

 **repeat**

  Generate the candidate frequent itemset (i.e., ship deficiency itemset candidates).

  *m* is the number of ship deficiency itemset, which will be updated by new candidate frequent itemsets.

  *n* is the transaction number in the PSC dataset, which is iteratively updated in the calculation procedure.

  *k* is the order of the candidate frequent itemset, and *k* is a positive number.

  **for** *i* = 1 to *m* **do**

   **for** *j* = 1 to *n* **do**

    Obtain the ship deficiency number for the candidate frequent itemset.

    Update the dataset by deleting the newly added transactions to calculate the support level.

   **end for**

  **end for**

  Calculate the support of the candidate frequent itemset using Eq (2).

  **if** *k* = 1 **do**

   select a frequent itemset based on *α*.

  **else if** *k* > 1 **do**

   Calculate the confidence value of the candidate frequent itemset using Eq (3).

   Select a frequent itemset based on *α* and *β*.

  **end if**

  Generate a new ship deficiency candidate frequent itemset by the adaptive connection mechanism.

 **until** the number of filtered-out frequent itemset *N* < 2.

**Fig 1:**
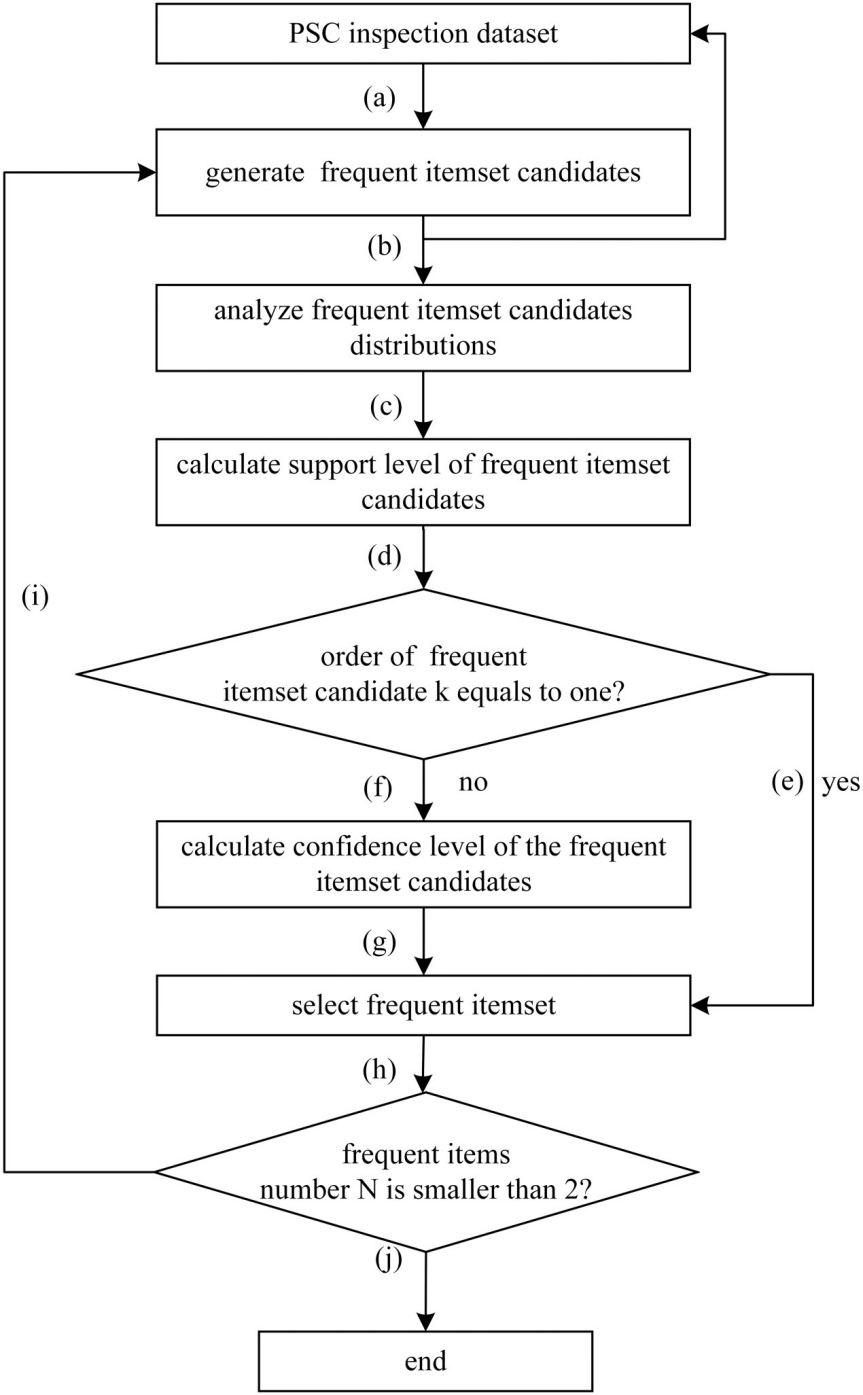
The schematic view of the proposed DQCPEA.

The specific DQCPEA procedure is consecutively implemented in the following steps (see Fig 1), which are explained in details as follows:

Step (a): Generate the candidate frequent itemset. The initial frequent itemset candidates (ship deficiency types) are deficiencies that have appeared in the PSC inspection data set. The frequent itemset candidates are generated from the last calculation results using the adaptive connection mechanism.
Step (b): Obtain the number of candidate frequent itemset (i.e., deficiency itemset candidates) and update the PSC inspection data set by mining the PSC inspection dataset. The data quality control procedure is implemented to update ship transaction data by suppressing the null ship inspection records to calculate the support level for each candidate ship deficiency. Note that the support level of the *k*th-itemset is an independent transaction when the itemset size is smaller than *k*. More specifically, we must set the candidate frequent itemset to (*k* + 1) to include the *k*th-itemset because the transaction for the ship deficiency itemset *k* does not include the *k*th-itemset. We consider the ship deficiency inspection data as invalid data when one of the following constraints is satisfied: (1) the ship deficiency category number inspected from a defective ship is less than the number of deficiencies in the *k*-itemset; (2) the deficiency number does not include itself (i.e., the *k*-itemset ship deficiency).
Step (c): Calculate the support level of the frequent itemset candidates according to Eq (2).
Step (d): Determine the number of items *k* in the frequent itemset candidates, which is the number of deficiencies in the deficiency itemset candidate.
Step (e): Select the frequent itemset according to the minimum support threshold *α* when *k* = 1.
Step (f): Calculate the confidence of the candidate frequent itemset according to Eq (3) when *k* > 1.
Step (g): Select the frequent itemset according to the minimum support threshold *α* and minimum confidence threshold *β*.
Step (h): Determine whether the filtered frequent itemset is empty.
Step (i): The model generates a new candidate frequent itemset by self-joining and performing a loop calculation when the number of frequent itemsets *N* is larger than 2. The adaptive connection mechanism is permutation combination of *k* frequent itemset to generating *k* + 1 candidate frequent itemset. For example, the candidate frequent itemset is set to $I_{2}^{\prime }=\{\left(a,b),(a,c),(b,a),(b,c),(c,a),(c,b\right)\},$ and the ship deficiency itemset *k* = 2 when frequent itemset *I*_1_ = {*a*, *b*, *c*} and *k* = 1. Similarly, the newly frequent itemset candidate is updated as $I_{3}^{\prime }=\{\left((a,b),c),((a,c),b),((b,c),a\right)\}$ and *k* = 3 when the ship frequent itemset is *I*_1_ = {(*a*, *b*), (*a*, *c*), (*b*, *c*)} and *k* = 2, respectively.
Step (j): The algorithm will terminate when the number of frequent items *N* is smaller than 2. The final outputs are the support level, itemset frequency and confidence level, which support the ship deficiency category identification.

## 3. Experiments

We employ the proposed DQCPEA framework to obtain the intrinsic correlation between the ship relevant factors (i.e., ship type, age, deadweight and gross tonnage, etc.) and the ship deficiency category (i.e., escape trunk blocked, statutory certificate expired, etc.) by mining the historical PSC inspection data. First, we explored the relationships between the ship relevant factors and ship deficiencies using quantitative analysis; then, the DQCPEA framework was introduced to find the implicit connections among various ship deficiency categories.

### 3.1. Dataset

The datasets are extracted from the Asia-Pacific PSC inspection dataset (mainly from Australia, Japan, China, etc.) within five years (January 2014 to December 2018). We have collected the ship deficiency categories from 10322 inspected ships. Following the rule in the PSC Manual of Tokyo memorandum of understanding (MOU) [40], the original ship inspection data mainly consist of fundamental ship information and PSC inspection information. This rule is applicable to the latter analysis section without specification. More specifically, the static ship data including the ship name, IMO, gross tonnage, deadweight, etc. are stored for ship safety traceability (which are easily accessed by PSC officials, ship owners, ship crew, etc.). The PSC inspection information includes the inspection date, inspection departments, ship deficiency category, etc. We format the original PSC inspection dataset into a two-dimension data array; the first data array is a type of nested array. More specifically, the first data array (named as ship information array) is nested with ship basic information with six columns including the ship type, age, deadweight, gross tonnage, detention state, and the second data array consists of the ship deficiency (named the ship deficiency array). The trivial PSC inspection data (e.g., certificate issuing office, call sign, inspection location) are discarded from the initial ship inspection data. The deficiency array shows specific ship deficiency categories that lead to ship detention results, and the ship information array contains the ship type, age, deadweight, and gross tonnage, which are considered the main factors that trigger a ship deficiency. Note that the data supporting the findings of this study are available within the article supplementary materials.

### 3.2. Ship deficiency analysis

The ships in similar states during the PSC inspection procedure may result in different detention results due to PSC subjective factors. More specifically, PSC officials in one location may observe several ship deficiencies types (which are not considered nonfatal factors) and do not implement further rectifications for the current voyage. A similar ship deficiency may be considered a fatal ship deficiency by PSC officials at other places. Previous studies attempted to suppress such inconsistent inspection results by determining crucial factors that trigger potential ship deficiencies, which are the ship type, age, gross tonnage and deadweight [39,41,42]. First, we qualitatively obtained the main factors from ships of triggering ship detention events with the historical PSC dataset; then, a quantitative analysis was implemented to determine the mutual correlations among different ship deficiency types. More specifically, we analyzed the ship detention cases through four factors to suppress trivial ship deficiencies (i.e., removing trivial ship deficiencies leading to ship detention events). Then, we quantitatively analyzed the intrinsic correlations among the ship deficiencies and the PSC reported ship detention events. According to the rule of thumb, we introduce six statistical indicators to quantitatively analyze the relationship ship deficiency triggering factors: ship type percentage (Stp), deficiency percentage (Def), detention percentage (Det), average deficiency number (Ave-Def), average detention number (Ave-Det), and deficiency number per detention (Def/Det). The ship type percentage reveals the ship deficiency distributions on different ship types.

#### 3.2.1 Ship type deficiency analysis

We have many ship type classifications due to different classification criteria. More specifically, we can obtain ship types according to the ship navigation area, power plant, etc. For example, the ships can be classified into ocean and offshore navigable categories. The ocean ships can implement the task of transmitting products in long range, while the offshore ships are permitted to sail in coastal areas. In our study, we divided the ship type based on ship usage purpose, and we collected 23 ship types (e.g., oil tanker, gas carrier, and passenger ship) from the PSC inspection dataset. The ship classification results are shown in Table 1.

**Table 1:** Ship type classifications and corresponding code IDs.

code ID*	ship type	code ID	ship type
311	NLS Tanker	363	woodchip carrier
312	combination carrier	367	livestock carrier
313	oil tanker	370	Ro-Ro passenger ship
320	gas carrier	371	passenger ship
323	commercial yacht	375	heavy load carrier
330	chemical tanker	376	offshore service vessel
340	bulk carrier	380	MODU & FPSO
352	vehicle carrier	382	special purpose ship
353	container ship	383	high speed passenger craft
355	Ro-Ro cargo ship	385	tugboat
360	general cargo/multi-purpose ship	399	other types of ship
361	refrigerated cargo carrier		

*The code ID is digital form for the corresponding ship type

**Fig 2:**
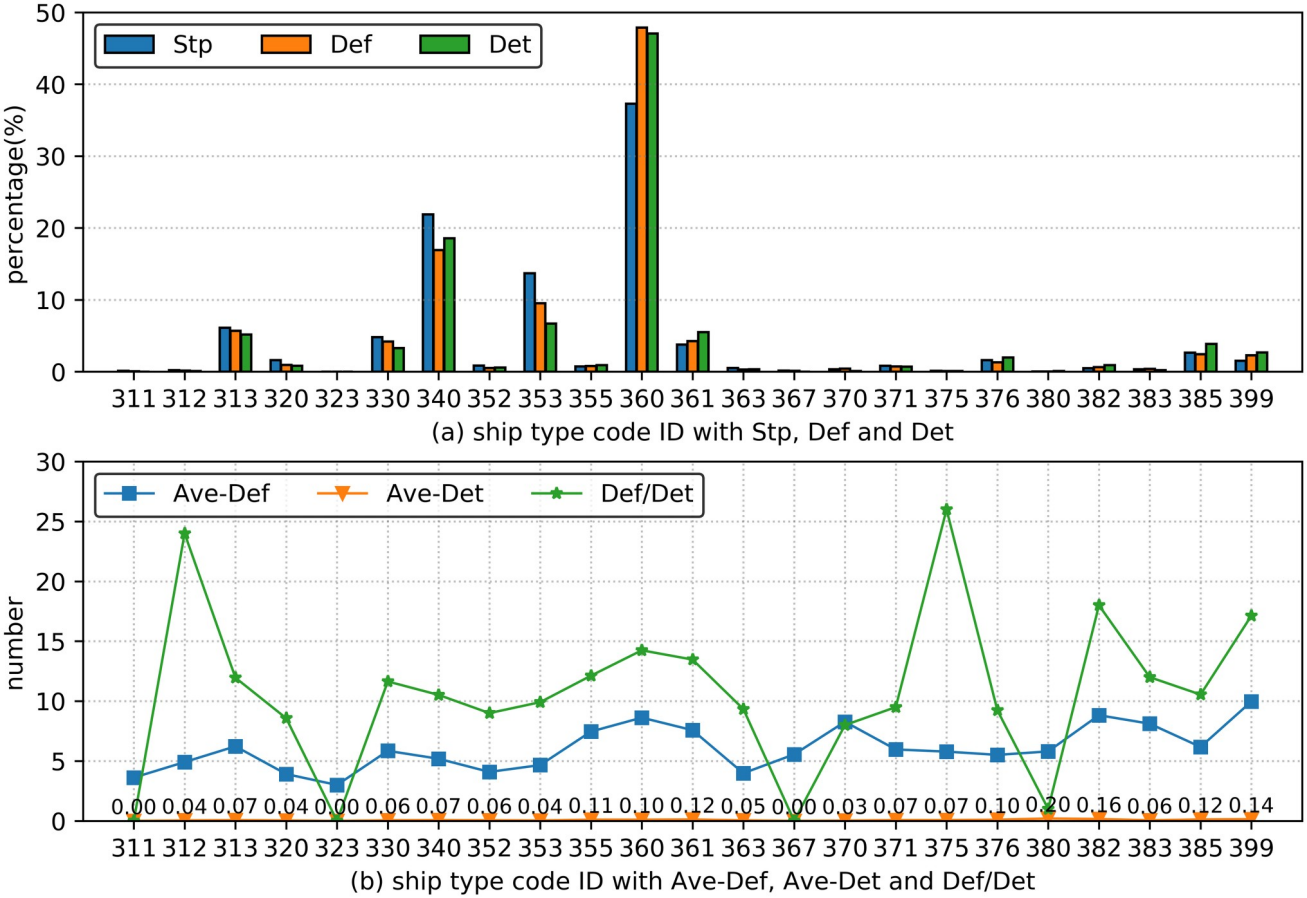
Ship type deficiency analysis. (a) The histograms show Stp, Def and Det under 23 different ship types. (b) The curves show Ave-Def, Ave-Det and Def/Det under 23 different ship types.

The ship deficiency analysis with ship type information reflects the relevance between different ship types and deficiency (as shown in Fig 2). Fig 2(a) indicated that general cargo/multi-purpose ship, bulk carrier and container ship (code IDs are 360, 340 and 353) are the top three ship detention types in terms of Stp, Def and Det. More specifically, the Stp indicators of the three ship types is 72.90%, which implies that general cargo/multi-purpose ship, bulk carrier and container ship are much more likely to be detained. The Def and Det indicators showed similar results to the Stp indicators: their values are 74.36% and 72.35%, respectively.

Fig 2(b) showed the distribution of three evaluation indicators (Ave-Def, Ave-Det and Def/Det) for different ship types. From the perspective of Ave-Def, the ship types with code IDs 399 and 382 (which are other types of ships and special purpose ship) are more prominent with Ave-Def values of 9.97 and 8.82, respectively. Thus, the ship types with code IDs 399 and 382 are likely to contain more deficiencies. After carefully reviewing the ship information from the initial PSC inspection data, we found that the ship type with code ID 399 consists of many small but in-poor-status ships, and the special purpose ships were inspected in a relatively more rigorous criterion. Meanwhile, the ship types with code ID 380 (mobile offshore drilling unit & floating production storage offloading, MODU&FPSO) and 382 (special purpose ship) ranked first and second in terms of Ave-Det, which were 0.20 and 0.16, respectively. This result demonstrated the intrinsic relationships between the deficiency characteristics and the potential ship detention rate. More specifically, the two types of ships with code IDs 380 and 382 are more easily detained than other ship types under similar inspection situations because these two ship types (MODU & FPSO and special purpose ship) are operated with higher requirements to safely transmitting goods. The Def/Det indicator distributions are significantly different from the ship type percent distribution: the minimum Def/Det is 0, while the maximum value is 26 (the values are adjusted according to the round-up criterion).

#### 3.2.2 Ship age deficiency analysis

**Fig 3:**
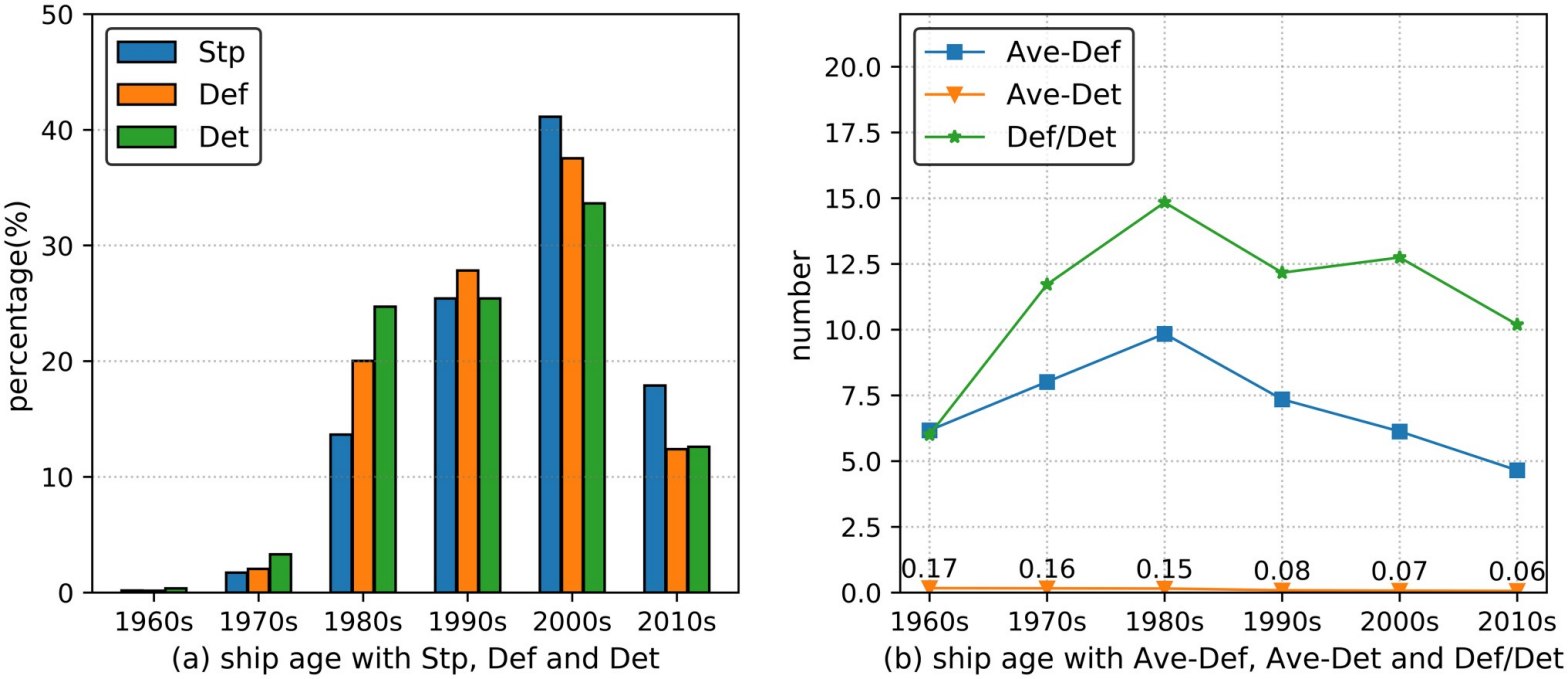
Ship age deficiency analysis. (a) The histograms show Stp, Def and Det within 1960s to 2010s. (b) The curves show Ave-Def, Ave-Det and Def/Det within 1960s to 2010s.

The ship age presents the ship current conditions, and potential ship deficiency characteristics can be obviously determined from the ship age information. We employed the keel laid date to estimate the ship age, and the ship building date samples were 1965–2017 in the PSC inspection dataset. We divided the ship building date samples into six groups (ranging from 1960s to 2010s), and the time interval was 10 years (as shown in Fig 3). Fig 3(a) showed that the decrease in Stp indicator from 1980s to 2010s occupied the vast majority of ship deficiency, since the longest ship in-service period is 40 years. The ships with longer service ages (i.e., more than 40 years) are inspected with stricter criteria before they are allowed to sail in the sea; thus, the ship ages in the time interval of 1960s-1970s only occupied a small proportion of ship detention samples.

Similarly, the Def and Det indicators account for a vast majority in the normal service period because of the ship number distributions, and the corresponding values are 97.79% and 96.35%, respectively. As shown in Fig 3(b), the curves of Ave-Def and Def/Det increased and decreased with the decrease in ship age (i.e., increase of manufacture date), and the maximum values were achieved in the 1980s. Hence, ships with longer service time are more likely to be inspected with deficiencies, which is considered the main reason for the maximal Ave-Def and Def/Det values for ships built in the 1980s. In addition, the Ave-Det curve in Fig 3(b) showed a downward variation tendency. The main reason is that the ship safety state is likely to deteriorate when the ship is old, so the ship is more likely detained during the PSC inspection procedures.

#### 3.2.3 Deadweight deficiency analysis

**Fig 4:**
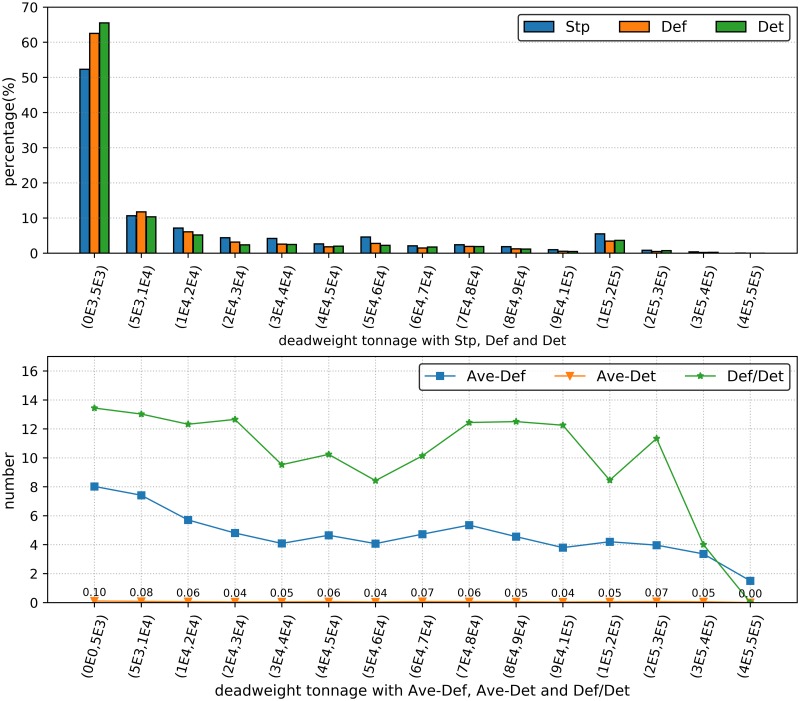
Ship deadweight deficiency analysis. (a) The histograms show Stp, Def and Det under different levels of deadweight. (b) The curves show Ave-Def, Ave-Det and Def/Det under different levels of deadweight.

The deadweight is the maximum operation tonnage that a ship can afford to carry goods. It can be used to estimate the ship trafficking capacity, and it is the basis for estimating the cost of ship renting and building. The maximum ship deadweight is not larger than 500,000 tons (t), and we divided the ship tonnage dataset into 15 groups to evaluate the deficiency analysis (as shown in Fig 4). It is noted that over 50% of the ship deadweight samples for the indicators Stp, Def and Det fall in the interval of 0–5,000 t, and a small proportion of the ship samples is 5,000–500,000 t (see Fig 4(a)). Overall, the three indicators Stp, Def and Det tended to decrease when the deadweight increases. However, the indicator values tended to increase when the deadweight is 100,000–200,000 t. More specifically, the ship type with 100,000–200,000 t mainly consists of oil tanker (i.e., the transmitting product is petroleum), which indicates that more attention is deserved to ensure the safety operation for the oil tanker. Fig 4(b) showed that Ave-Def. Ave-Det and Def/Det tended to increase and subsequently decrease, and the maximum values were obtained for 0–5,000 t. Such ships are mainly small cargo ships and are likely detained after being inspected to reduce maritime traffic risk.

#### 3.2.4 Gross tonnage deficiency analysis

**Fig 5:**
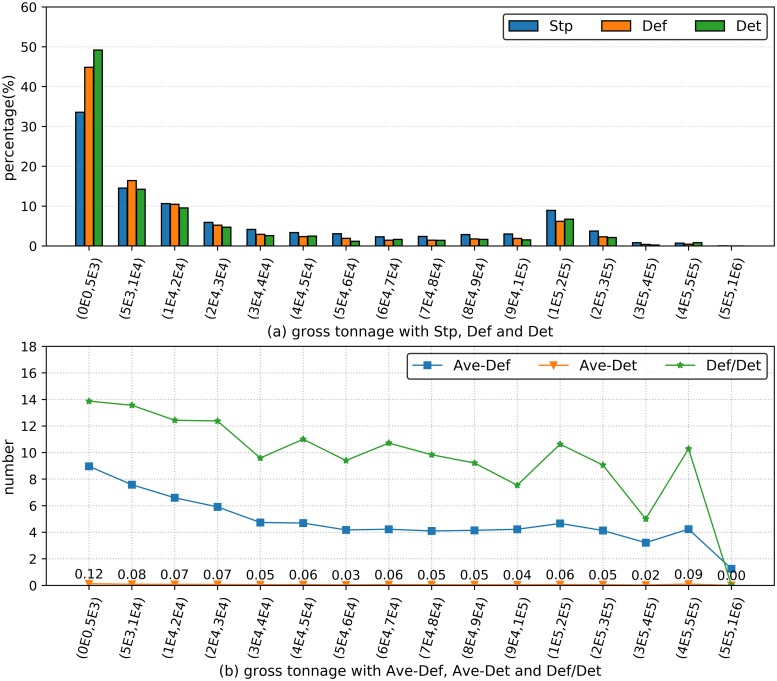
Ship gross tonnage deficiency analysis. (a) The histograms show Stp, Def and Det under different levels of gross tonnage. (b) The curves show Ave-Def, Ave-Det and Def/Det under different levels of gross tonnage.

The gross tonnage is the total capacity of the entire ship calculated according to certain criteria issued by the ship classification society, which is known as the total volume. It is usually used to indicate the size of the ship, calculate the cost of building and insurance, etc. Based on the collected PSC inspection dataset, the maximum gross tonnage of the ship does not exceed 1000000 t, and 16 groups are classified according to the gross tonnage to analyze the defective characteristics (as shown in Fig 5). The indicator variation tendency in Fig 5 is quite similar to that in Fig 4. More specifically, the Stp, Def and Det indicators tended to decrease when the deadweight increases (as shown in Fig 5(a)), which reached the peak value when the tonnage is 100,000–200,000 t. The Ave-Def curve in Fig 5(b) showed a downward trend when the gross tonnage increases, which is indeed considered a positive correlation to the ship type percentage under different gross tonnages. Moreover, the Ave-Det and Def/Det curves tended to increase and subsequently decrease, and the maximum value was achieved in the tonnage interval of 400,000–500,000 t, while the trough was obtained at 300,000–400,000 t.

### 3.3. DQCPEA analysis

The previous section qualitatively analyzes the correlation between ship state information and ship deficiencies. Using the DQCPEA model, we quantitatively explored this relationship. We divided the ship defective types into 18 types of deficiency categories, which were further classified into 568 ship deficiency subcategories. Each ship deficiency subcategory is assigned a code ID. More specifically, each ship deficiency is encoded by a two-digit ID (which is named the parent ship deficiency), while the corresponding subcategory is presented with a five-digit ID (the former two digits are the parent ship deficiency code ID). According to the rule of thumb, there is a ship deficiency category correlation when the ship subcategory correlations occur. Thus, we set different minimum support level thresholds *α* and minimum confidence level thresholds β to implement a holistic analysis on the correlation exploration on the parent ship deficiency category; then, we fine-tuned the parameter settings to further explore the subcategory ship deficiency correlations. The parameter setups to analyze the parent ship deficiency correlations are as follows: (1) *α* = 0.2, β = 0.5; (2) *α* = 0.25, β = 0.5; (3) *α* = 0.20, β = 0.6; (4) *α* = 0.25, β = 0.6. The combination settings to explore the subcategory ship deficiency correlations are as follows: (1) *α* = 0.1, β = 0.5; (2) *α* = 0.15, β = 0.5; (3) *α* = 0.1, β = 0.6; (4) *α* = 0.15, β = 0.6.

#### 3.3.1 Correlation analysis on the parent ship deficiency category

The parent ship deficiency categories include all factors that pose a significant risk to the maritime safety and ship crew living and working conditions on board. The parent ship deficiency category in our study does not involve the ship deficiency location. Detailed information about the specific ship deficiency code IDs and specific defective items is shown in Table 2.

**Table 2:** Parent ship deficiency code IDs and corresponding defective items.

code ID	defective item	code ID	defective item
01	certificate & documentation	10	safety of navigation
02	structural conditions	11	lifesaving appliances
03	water/weathertight conditions	12	dangerous goods
04	emergency systems	13	propulsion and auxiliary machinery
05	radio communications	14	pollution prevention
06	cargo operations including equipment	15	ISM
07	fire safety	16	ISPS
08	alarms	18	labor conditions
09	working and living conditions	99	other

The correlation among different deficiency categories were obtained using the DQCPEA model under different minimum support thresholds *α* and minimum confidence thresholds *β*. Table 3 shows the results of the frequent item sets in different threshold combination scenarios. We found that the frequent itemset contained few ship deficiencies items when thresholds *α* and *β* are very large. The reduction of the threshold settings provided us more reasonable ship deficiency correlation explorations results. We also found that code ID 01 (which is certification & documentation) is the most frequent item under different *α* and *β* setting combinations, which indicates that the ship deficiency with code ID 01 (certification & documentation) is the most common deficiency category in the ship inspection procedure.

**Table 3:** Frequent itemsets exploration under different combinations of *α* and *β* for the parent ship deficiency analysis.

*α*, *β*	frequent items
0.20,0.5	(03,01), (04,01), (07,01), (10,01), (11,01), (14,01), (15,01), (18,01), (07,10), (10,07), (07,11), (11,07), (10,11), (11,10), ((01,07),10), ((01,07),11), ((01,10),07), ((01,10),11). ((01,11),07), ((01,11),10), ((07,10),01), ((07,11),01), ((10,11),01)
0.25,0.5	(03,01), (04,01), (07,01), (10,01), (11,01), (14,01), (15,01), (18,01), (07,10), (11,07), (11,10), ((01,07),10), ((01,11),07), ((01,11),10), ((07,10),01), ((07,11),01), ((10,11),01)
0.20,0.6	(03,01), (07,01), (10,01), (11,01), (07,10), (10,07), ((01,07),10), ((01,10).07), ((07,10),01)
0.25,0.6	(03,01), (07,01), (10,01), (11,01), ((01,07),10), ((07,10),01)

**Fig 6:**
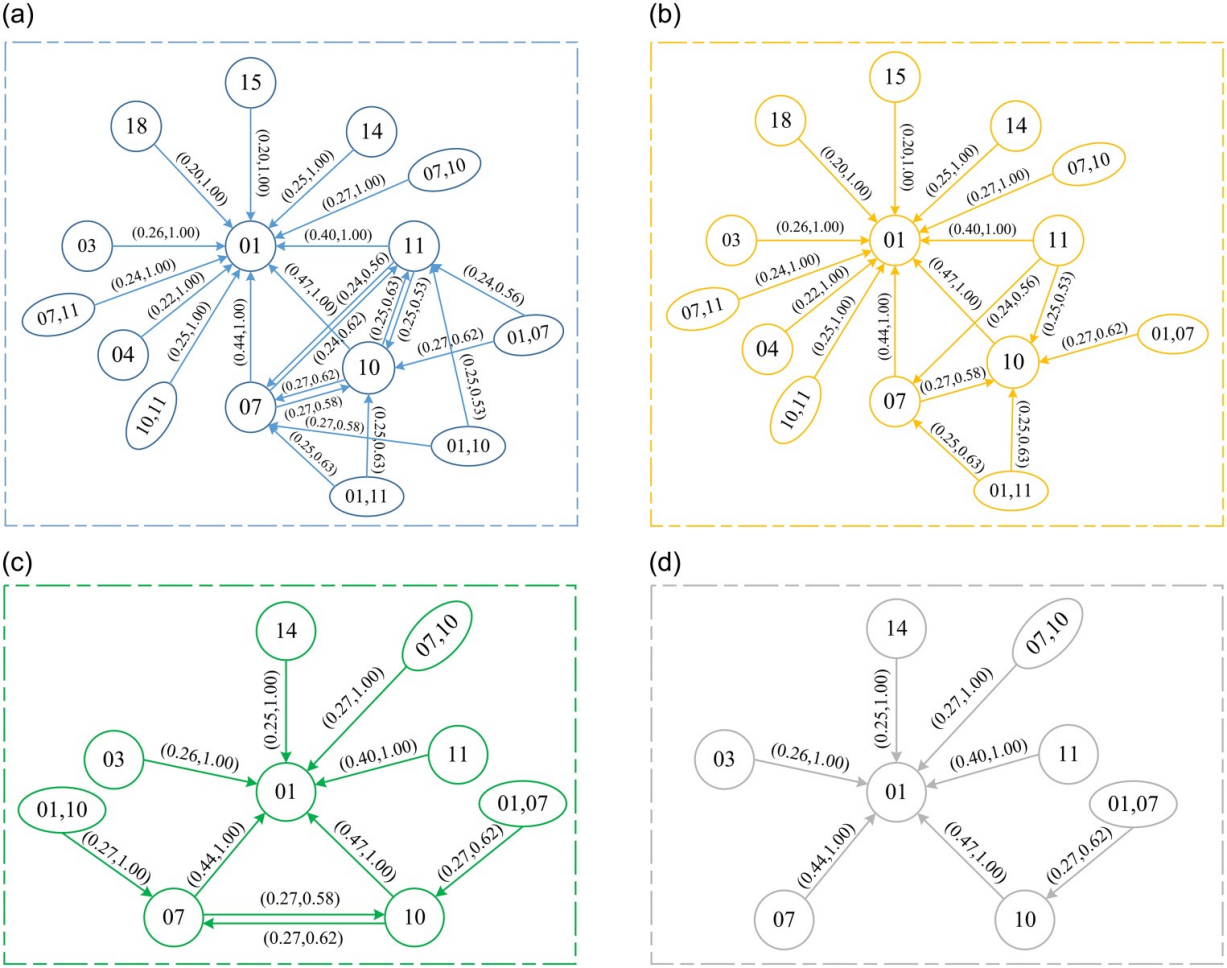
Correlation exploration network of items of frequent itemsets in parent deficiency category with different *α* and *β* setups. (a) *α* = 0.20, *β* 0.5. (b) *α* = 0.25, *β* = 0.5. (c) *α* = 0.2, *β* = 0.6. (d) *α* = 0.25, *β* = 0.6.

Fig 6 presented the correlation network among different items of frequent itemsets under different combinations of *α* and *β*. Overall, the smaller settings of thresholds *α* and *β* result in very complicated correlation networks, while the complex correlation network contains basic correlations networks (which are obtained by setting larger *α* and *β*). In addition, Fig 6 presented the correlation level (i.e., values of support and confidence) among different frequent items. For example, code IDs 01 (certificate & documentation), 07 (fire safety) and 10 (safety of navigation) simultaneously occur with a probability of 0.27. More specifically, ship deficiencies with code IDs 01 and 07 simultaneously occur, which makes the deficiency occurrence of code ID 10 being 0.27. In addition, we observed that the probability of determining ship deficiency with code ID 10 (safety of navigation) is 0.62 (i.e., the confidence is 0.62) when the ship deficiencies certificate & documentation and fire safety simultaneously occur.

#### 3.3.2 Correlation analysis of deficiency subcategories

The correlation analysis on deficiency categories provides basic guiding rules for ship deficiency inspection. It is not easy to explore holistic ship deficiencies by exhaustively searching. The PSC inspection data suggested that ship crew certificate maybe expired (i.e., staff working on-board may not be qualified), and thus impose threat to maritime traffic safety. The historical PSC inspection data have shown that navigation safety relevant issues were considered as another crucial factor leading to many ship detain events, and thus addressing such issues can significantly improve waterway safety. We obtained 568 ship deficiency subcategories based on the parent ship deficiency category classifications. Table 4 shows a specific classification that the number of deficiency subcategories belongs to each parent ship deficiency category. We find that the certificate & documentation subcategory occupied the largest number in the parent deficiency category, which confirms our aforementioned analysis, and the minimal ship deficiency subcategory has code ID 99.

**Table 4:** The details of deficiency subcategory classification.

code of deficiency category	01	02	03	04	05	06	07	08	09
number of deficiency subcategories	100	33	15	19	18	10	26	11	73
code of deficiency category	10	11	12	13	14	15	16	18	99
number of deficiency subcategories	36	36	10	9	76	13	7	73	3

Table 5 shows the results of frequent item sets under combinations of different thresholds in the deficiency subcategory correlation analyzed by the proposed DQCPEA model. The support level significantly affects the number of items of the frequent item sets, and its effect on the confidence level is quite limited. Table 5 shows that the difference in number of items of the frequent item sets is very small when the parameter settings are set as *α* = 0.5, *β* = 0.6 and *α* = 0.1, *β* = 0.6. Meanwhile, the frequent item sets are the same when the parameter settings are *α* = 0.15, *β* = 0.5 and *α* = 0.15, *β* = 0.6. The defective subcategory codes and corresponding defective items in the frequent item sets are presented in Table 6.

**Table 5:** Frequent itemsets exploration under different combinations of *α* and *β* for deficiency subcategory correlation analysis.

*α*, *β*	frequent items
0.10, 0.5	(07199,01214), (10109,01214), (10111,01214), (10116,01214), (11101,01214), (01308,01220), (07105,01220), (07199,01220), (10109,01220), (10111,01220), (10116,01220), (10109,10111), (10116,10111), (10111,10116), (10127,10111)
0.10, 0.6	(10109,01214), (10111,01214), (11101,01214), (01308,01220), (07105,01220), (07199,01220), (10109,01220), (10111,01220), (10116,01220), (10116,10111), (10127,10111)
0.15, 0.5	(10111,01214), (10111,01220)
0.15, 0.6	(10111,01214), (10111,01220)

**Table 6:** Ship deficiency subcategory code IDs and corresponding defective items.

code ID	defective item	code ID	defective item
01214	endorsement by flag state	10109	lights, shapes, sound-signals
01220	seafarers’ employment agreement (sea)	10111	charts
01308	records of rest	10116	nautical publications
07105	fire doors/openings in fire-resisting divisions	10127	voyage or passage plan
07199	other (fire safety)	11101	lifeboats

**Fig 7:**
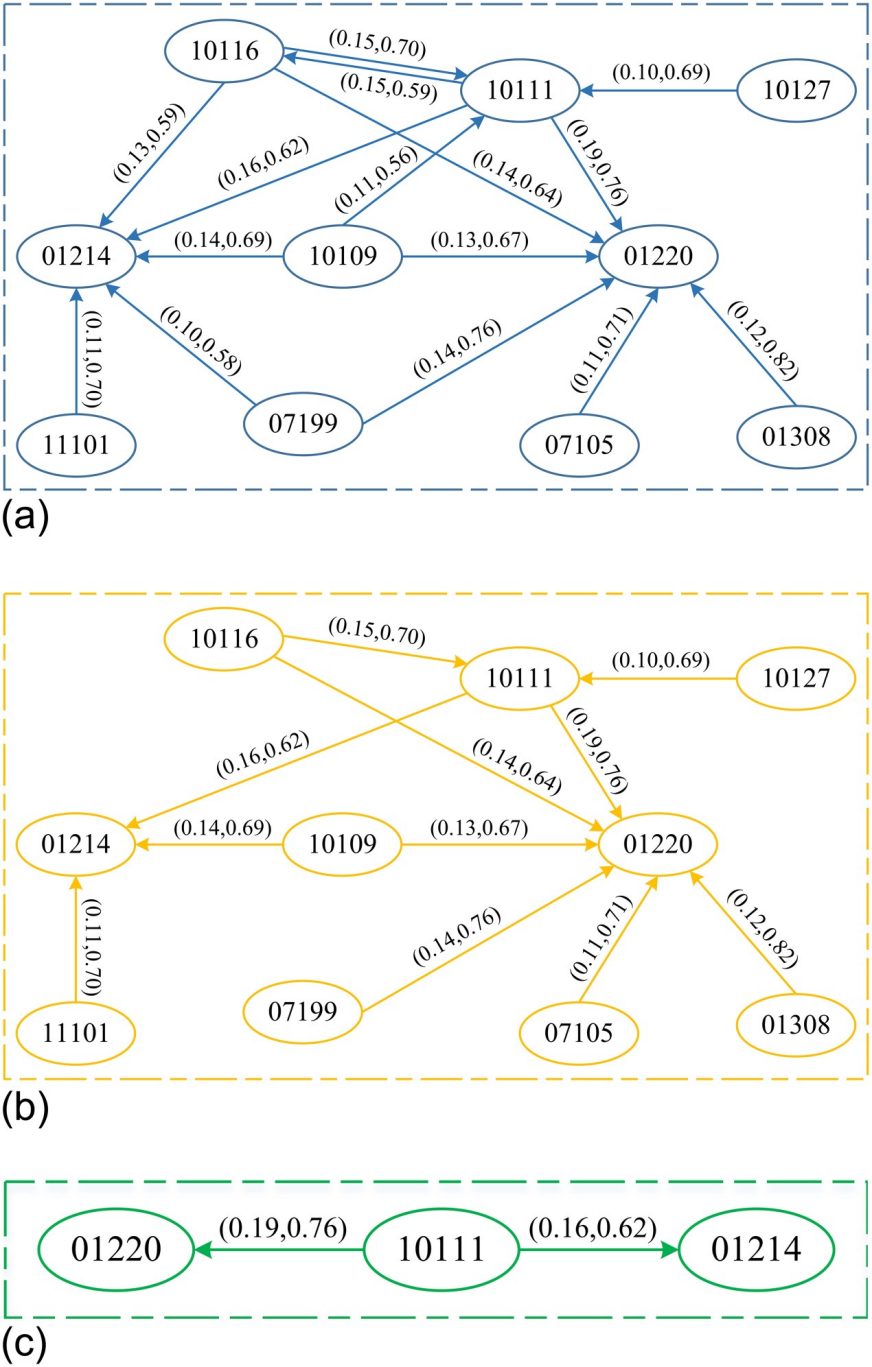
Correlation exploration network of items of frequent itemsets in ship deficiency subcategories with different *α* and *β* setups. (a) (*α* = 0.10, *β* = 0.5). (b). (*α* = 0.10, *β* = 0.6). (c) (*α* = 0.15, *β* = 0.5) or (*α* = 0.15, *β* = 0.6).

Fig 7 illustrated the correlation among different frequent items at different combination settings of *α* and *β*. In general, the analyzed deficiency subcategory has fewer items of frequent item sets than the analyzed deficiency category, and the correlation networks are considered much simple. We cannot find 3 items in the frequent item sets because the deficiency is presented in a more detailed and classified manner, which makes the ship deficiency subcategories irrelevant. As shown in Fig 7, the endorsement by flag state (code ID is 01204) and seafarers’ employment agreement (code ID is 01220) have higher probabilities of deficiency, and there are two ship deficiency categories with many other deficiencies. For example, we observe that the probability of simultaneous occurrence of deficiencies 01220 (Seafarers’ employment agreement, SEA) and 10111 (Charts) is 0.19 (the support is 0.19), and the probability of occurrence of deficiency 01220 (Seafarers’ employment agreement, SEA) is 0.76 (the confidence is 0.6) in the case of deficiency 10111 (Charts).

## 4. Conclusions

The correlation analysis on the ship deficiencies from the PSC inspection dataset provides crucial ship inspection guidelines, which can identify the potential maritime traffic risking factors and significantly improve maritime safety. Using the proposed DQCPEA framework, we have conducted a comprehensive ship deficiency correlation analysis by deeply diving into the PSC inspection dataset (i.e., focused on the ship type, age, deadweight and gross tonnage). The ship type percentage, deficiency percentage, detention percentage, average deficiency number, average detention number and deficiency number per detention were introduced to quantitatively analyze the ship deficiency correlations. The experimental results indicate that the ship type, age, deadweight and gross tonnage are closely related to the ship deficiency identification, which is particularly obvious in the ship detention incidents. We also found internal correlations among defective categories and defective subcategories. The correlation analysis of the defective categories provides the PSC relevant participants with theoretical guiding rules, which may impose a limited effect on the PSC inspection practices, since the ship deficiencies are too generalized to rectify. Meanwhile, the correlation analysis of defective subcategories provides the basis for the PSC inspection considering that the ship subcategory deficiencies are easily understandable and rectified by the ship crew.

Although the proposed DQCPEA framework provides us satisfactory ship deficiency correlation analysis results, we can expect to extract more intrinsic ship deficiency correlations in the following aspects. First, the support level among the ship deficiency frequent items can be further enhanced because the correlation is explored from the PSC inspection data from Tokyo MOU. In the future, we can enlarge the PSC inspection dataset by incorporating additional PSC data (e.g., data from Paris MOU and United States Coast Guard). Second, we can explore our research by developing graph-based knowledge discovery models to help PSC relevant participants understand the ship deficiency evolution spectrum. Third, testing and evaluating our model performance against other models can further demonstrate the proposed framework superiority.
